# Effect of Laser Irradiation Modes and Photosensitizer Types on Antimicrobial Photodynamic Therapy (aPDT) for *Streptococcus sobrinus* in the Crown Dentin of Bovine Teeth: An Experimental In Vitro Study

**DOI:** 10.3390/dj12030059

**Published:** 2024-03-01

**Authors:** Yohei Yamaguchi, Daiki Yoshii, Hiroaki Katsuragi, Koichi Shinkai

**Affiliations:** 1 Advance Operative Dentistry-Endodontics, The Nippon Dental University Graduate School of Life Dentistry at Niigata, 1-8 Hamauracho, Chuo-ku, Niigata 951-8580, Japan; yohfey@ngt.ndu.ac.jp; 2 Department of Operative Dentistry, The Nippon Dental University School of Life Dentistry at Niigata, 1-8 Hamauracho, Chuo-ku, Niigata 951-8580, Japan; yoshii.daiki@ngt.ndu.ac.jp; 3 Research Center for Odontology, The Nippon Dental University School of Life Dentistry, 1-9-20 Fujimi, Chiyoda-ku, Tokyo 102-8159, Japan; katsura@ngt.ndu.ac.jp

**Keywords:** antimicrobial photodynamic therapy, laser irradiation modes, photosensitizer types, *Streptococcus sobrinus*, infected dentin plate

## Abstract

This study aimed to assess the impact of different laser irradiation modes and photosensitizer types on the bactericidal efficacy of antimicrobial photodynamic therapy (aPDT). Dentin plates were prepared by sectioning the crown dentin of bovine teeth infected with *Streptococcus sobrinus* (n = 11). Nine aPDTs involving the combination of three 1% solutions of photosensitizers (brilliant blue, BB; acid red, AR; and methylene blue, MB) and three irradiation modes of semiconductor lasers (50 mW for 120 s, 100 mW for 60 s, and 200 mW for 30 s) were performed for each infected dentin plate, and the control consisted of the specimens not applied with aPDT. The bactericidal effects in 10 groups were evaluated using both assays of the colony count (colony-forming-unit: CFU) and adenosine triphosphate (ATP) (relative-light-unit: RLU). The data obtained were analyzed using the Kruskal–Wallis test (α = 0.05). The most aPDT groups exhibited significantly lower RLU and CFU values compared with the control (*p* < 0.05). The effect of irradiation modes on RLU and CFU values was significant in the aPDT group using BB (*p* < 0.05) but not in the aPDT group using AR or MB. The aPDT performed with AR or MB exerted a remarkable bactericidal effect.

## 1. Introduction

Antimicrobial photodynamic therapy (aPDT) is known to eliminate bacterial infectious agents using reactive oxygen species (ROS) generated by irradiating a photosensitizer (PS) with a light source, particularly laser, of the same excitation wavelength. In the field of dentistry, aPDT is currently being studied for use to treat periodontal diseases [1,2,3], endodontics [4,5], and peri-implantitis [3,6,7], as well as dental caries [8,9,10]. The mechanism of action of aPDT is that, when PS is excited by laser irradiation at a specific wavelength, it forms an excited triplet state, which transfers energy to oxygen molecules to form highly cytotoxic ROS such as hydroxyl radicals and singlet oxygen [11]. ROS alter bacterial cell membrane structure [12] and cause cell death through several mechanisms important for the maintenance of biological systems [13].

In routine clinical caries treatment, dentin caries extending close to the pulp may be encountered. In such cases, it is essential to prioritize pulp preservation and avoid exposure of the pulp as much as possible by applying temporary indirect pulp capping (IPC) [14,15]. However, IPC requires a complicated procedure and a long treatment period before the final restoration. In the past, capping over carious dentin with pulp capping materials containing antimicrobial agents was attempted [16], whereas this method was not popular in clinical practice due to concerns about the emergence of resistant bacteria. In recent years, a treatment method that intentionally leaves only the carious dentin near the pulp and capping it with bio-inductive material has been attempted in clinical practice. [14]. However, the bactericidal effect of bio-inductive materials on cariogenic bacteria is unknown because there was no report examining the antimicrobial properties of bio-inductive materials. Meanwhile, the bactericidal effect of aPDT on cariogenic bacteria has attracted much attention, and many basic and clinical studies have been conducted [17,18,19]. Although the results of these studies all suggested that aPDT was effective in the treatment of dental caries, the kinds of photosensitizers and types and wavelengths of light sources used in these studies varied widely. Moreover, most of the basic studies that investigated the bactericidal properties of aPDT against cariogenic bacteria have been conducted on agar media, and a few studies mimicked caries lesions [20,21]. Therefore, the further accumulation of basic data must be required.

The clinical application of aPDT for caries treatment, particularly in the dentin, requires consideration of complex morphologies, such as dentinal tubules [22,23]. Therefore, in vitro studies of aPDT should be conducted under simulated clinical conditions. Using infected bovine dentin plates, Nagai et al. [20] and Yoshii et al. [21] demonstrated the bactericidal effect of aPDT against *Streptococcus mutans* (*S. mutans*) and *Lactobacillus acidophilus* (*L. acidophilus*), respectively. Although these bacteria are the primary cariogenic bacteria, it is well known that other species can also be detected in dentin caries. *Streptococcus sobrinus* (*S. sobrinus*) is widely recognized as a major cariogenic bacterium along with *S. mutans* and *L. acidophilus* [24,25,26]. Characteristics of *S. sobrinus* compared to *S. mutans* include less detection in saliva but more acid production [27], different mechanisms of acid tolerance response [24,28], immunosuppression [29], hydrogen peroxide production [24], and the ability to increase glucan synthesis [24]. Okada et al. [30] reported that schoolchildren carrying both *S. mutans* and *S. sobrinus* in their oral cavity had significantly higher incidence of caries in both permanent and primary teeth compared with those carrying only *S. mutans*, suggesting that *S. sobrinus* plays an important role in caries development and severity. In addition, Korona-Glowniak et al. [31] reported the correlation between the dissemination rate of *S. sobrinus* and the prevalence of dental caries. Therefore, it is important to evaluate the bactericidal effect of aPDT against *S. sobrinus*. However, in vitro studies focusing on the above topic are scarce and are mostly conducted on agar medium or plastic plates; thus, a more clinically oriented evaluation of infected dentin is preferable.

When using laser irradiation with a wavelength corresponding to the excitation wavelength of PS to which it is applied, aPDT is effective. Various combinations of PS and lasers have been investigated; however, no clear standards for laser irradiation time or power have been established yet, and different researchers used different irradiation protocols even if the PS and laser modes were the same. Furthermore, the optimal conditions for laser irradiation in aPDT have not yet been set up in experimental studies, and not all the parameters in different modes have been evaluated in review articles [17,18,32,33]. In addition, most of these studies involved only the parameters of irradiation time and power [34]. When caries treatment applying aPDT is clinically simulated, an increase in intrapulpal temperature due to laser irradiation is a concern. Studies that observed changes in intrapulpal temperature during laser irradiation reported that the temperature increase in the pulp was influenced by the total energy of the laser [35]. Therefore, in the present study, several laser irradiation powers and times were set in aPDT so that the amount of laser irradiation energy was identical.

In our series of studies on aPDT, we used various photosensitive agents, e.g., azulenocyanine (AC), methylene blue (MB), brilliant blue (BB), and acid red (AR). AC is stimulated by the laser with a wavelength of approximately 1000 nm [36]. MB has been popularly used with in vitro studies on the bactericidal effect of aPDT. In clinical practice, aPDT with MB is applied for sterilization of periodontal pockets. These photosensitizers exhibited bactericidal effects against *S. mutans* and *L. acidophilus* when used in aPDT [20,21]. The bactericidal activity of PS alone against cariogenic bacteria has been demonstrated in the papers by Nagai et al. and Yoshii et al. Nagai et al. found that MB showed some bactericidal effect against *S. mutans*, whereas the effect was significantly lower than the bactericidal effect of aPDT, which combines MB and laser irradiation [20]. Yoshii et al. also revealed that BB and AR showed little bactericidal effect when used alone against *L. acidophilus* [21]. BB and AR are components of caries-detecting solutions and can stain infected dentin [37]. These may be also applied to aPDT as a photosensitizer, because they release reactive oxygen species with laser light at a wavelength of 650 nm [21]. Therefore, both BB and AR are expected to be the photosensitizer used in aPDT for the sterilization of caries dentin. However, it is not clarified whether aPDT with these photosensitizers is effective in bactericidal action against *S. sobrinus*.

This study aimed to compare the bactericidal effect of aPDT in *S. sobrinus*-infected dentin plates with various PS and laser irradiation protocols, where the power and irradiation times varied so that the amount of irradiation energy remained constant. The null hypothesis was that “laser irradiation conditions and type of photosensitizer would not affect the bactericidal effect of aPDT on *S. sobrinus*”.

## 2. Materials and Methods

### 2.1. Preparation of Dentin Plates

Freshly extracted mandibular bovine teeth were purchased from Niigata Meat Plant (Niigata, Japan) and immediately stored in a freezer at −20 °C until usage. When used, the teeth were thawed naturally, and those showing cracks or significant occlusal wear were excluded. Dentin plates measuring approximately 3 × 3 × 1 mm were fabricated from the cervical parts of the crowns of extracted mandibular bovine teeth using IsoMet Low Speed (BUEHLER, Lake Bluff, IL, USA). A 40% phosphoric acid solution (K-etchant GEL, Kuraray Noritake Dental Inc., Tokyo, Japan) was applied to the dentin plate surface for 3 min for demineralization, followed by thorough rinsing with distilled water and ultrasonic cleaning using a machine (US Cleaner; As One, Osaka, Japan) for 5 min. Thus, the preparation of the dentin plates enabled easy bacterial invasion, which were then sterilized using an autoclave (2 atm, 121 °C, 15 min). The preparation of the dentin plates described above was carried out according to the method previously used in our laboratory [20,21].

### 2.2. Preparation of Bacterial Solution

**Figure 1:**
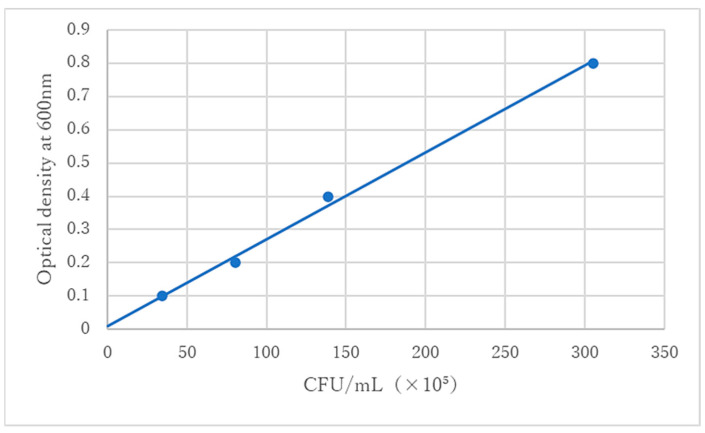
Optical density values were used to define the relevant concentration of bacterial solution. The bacterial cell counts of *S. sobrinus* at the optical densities of 0.1, 0.2, 0.4, and 0.8 are shown at the standard line. In this study, the concentration of the bacterial solution corresponded to the optical density of 0.3.

The *S. sobrinus* (ATCC33478) used in this study was isolated from human dental plaque. The *S. sobrinus* were immersed in sterile phosphate-buffered saline (PBS) (Fujifilm Wako Pure Chemical Industries Ltd., Tokyo, Japan), and the optical density of the obtained bacterial suspension liquid was adjusted to 0.3 at 600 nm based on the standard line (Figure 1). Optical density was measured using a spectrophotometer (Colourwave CO7500 Colorimeter; Biochrom, Cambridge, UK).

### 2.3. Preparation of Infected Dentin Plates

Dentin plates were placed one by one in the wells of a 24-well cell culture plate, and 20 µL of adjusted bacterial suspension was poured onto each of the dentin plates. The cell culture plate was centrifuged at 2000 rpm for 10 min to allow bacterial invasion of dentinal tubules. Then, 1.0 mL of brain heart infusion (BHI) (Benton, Dickinson and Company Inc, Franklin Lakes, NJ, USA) was added to each well, and the cell culture plate was incubated for 3 h at 37 °C in a 10% CO_2_ environment using an incubator (Shaking Mixer SHM-101, AGC Techno Glass Co., Ltd., Shizuoka, Japan). After incubation, the BHI was removed, and then 1.0 mL of 10 mM PBS was added to each well. The cell culture plate was shaken for 1 min using a rotary permeabilizer to eliminate as many bacteria outside the dentinal tubules as possible. This procedure was repeated three times. The preparation of the infected dentin plates described above was also carried out according to the method previously used in our laboratory [20,21].

### 2.4. Laser Irradiation Modes

The semiconductor laser used in this study was the P2 Dental Laser System (Pioon Laser Technology Co, Wuhan, China). First, the laser handpiece used for aPDT was attached to a flexible arm, and the position of the irradiation light tip (8 mm in diameter) was adjusted so that the irradiation distance was 10 mm. The wavelength, irradiation mode, and power of laser were set to 650 nm, CW, and 50 mW, 100 mW, and 200 mW, respectively. The irradiation times for the powers of 50, 100, and 200 mW were set to 120, 60, and 30 s, respectively. Under all the conditions, the total energy dose and density were set to 6 J and 11.9 J/cm^2^, respectively.

### 2.5. Adjustment of PS

Brilliant blue (BB) (Tokyo Chemical Industries Co., Ltd., Tokyo, Japan), acid red (AR) (Tokyo Chemical Industries Co., Ltd., Tokyo, Japan), and methylene blue (MB) (Fujifilm Wako Pure Chemical Industries Ltd., Tokyo, Japan) were used as the PS. Their concentration was adjusted to 10 mg/mL, or 1%, via dilution with PBS. Then, the PS solutions were sterilized via filtration with a 0.22 μm membrane filter (Merck Millipore Ltd., Tullagreen, Corrigtwahill, Co., Cork, Ireland).

The adjusted PS was stored in the refrigerator at 4 °C until the end of the experiment (within 50 days) to avoid the influence of light. Whenever PS was used, it was brought to room temperature and used only as needed.

### 2.6. Experimental Groups and aPDT Treatment

Table 1 presents the code for each aPDT experimental group. Nine aPDT experimental groups were formed by setting the respective combined irradiation parameters (50 mW × 120 s, 100 mW × 60 s, and 200 mW × 30 s) and PS (BB, AR, and MB). The untreated experimental group was defined as the control (cont). Power analysis was conducted using the G*power software (version 3.1.9.7; Franz Faul University, Kiel, Germany) with the effect size of 0.4 (Cohen’s large-effect size) power of 0.8 and a sample size of 110. As a result, the number of specimens in each group were 11 (n = 11).

Ten infected dentin plates were positioned in the wells of the 24-well cell culture plate, saturated with 200 µL of each PS, and then stored in a dark place for 5 min for pre-irradiation time. The plates were then irradiated with laser according to each irradiation parameter. The aPDT treatment described above was carried out according to the method previously used in our laboratory [20,21].

### 2.7. Detachment of Viable Bacteria from Infected Dentin Plate

After aPDT treatment, excess PS solution adhering to the dentin plate was washed off with PBS. A microtube containing 1.0 mL of PBS was set in an ultrasonic cleaner (US Cleaner; As One, Osaka, Japan), and the dentin plate was immersed in PBS with the dentin plate grasped with tweezers and sonicated for 3 min to detach *S. sobrinus* from the dentin plate. After the dentin plate grasped with tweezers was then pulled out from the PBS, the PBS containing bacteria was vortexed for 1 min to prepare a bacterial suspension. This bacterial suspension was used for an ATP assay and colony count assay.

**Figure 2:**
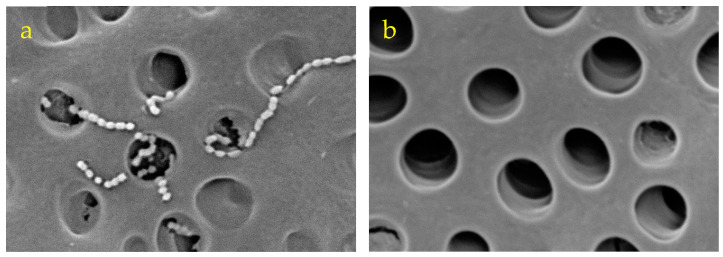
SEM images before (**a**) and after (**b**) treatment for bacterial detachment. *S. sobrinus* that entered the dentin tubular canal opening were completely removed with ultrasonic cleaning. Magnification: ×5000.

Separation of the bacteria from the dentin plate was confirmed via scanning electron microscopy (SEM) observation in a preliminary experiment. In the experiment, the infected dentin plates were immersed in 2.5% glutaraldehyde, fixed for 1 h at 4 °C, immersed in 70% ethanol overnight, and then subjected to stepwise dehydration (80%, 90%, and twice of 99%) in ethanol for 1 h per step. After the indicated process, they were degasified in a vacuum desiccator for 24 h, and then platinum for the SEM observation was deposited on their surfaces. The surface of the dentin plate was observed under high magnification at an acceleration voltage of 5 to 15 kV to confirm bacteria detachment using the TM4000 PLUS Miniscope (HITACHI High-Technologies Corp., Tokyo, Japan). Comparison of the SEM images before (Figure 2a) and after (Figure 2b) treatment for bacterial detachment showed that the bacteria were sufficiently separated and transferred from the dentin plate (Figure 2).

### 2.8. ATP Assay

An ATP assay was performed as follows. First, ATP was extracted from the bacterial suspension by mixing a 0.1 mL ATP extraction reagent (Toyo B-net Co., Tokyo, Japan) with a 0.1 mL bacterial suspension and leaving the mixture for 10 s. After adding 0.1 mL of Kinshiro^®^ ATP Luminescence Kit version 2 (Toyo B-net Co., Tokyo, Japan) to 0.1 mL of the ATP extract, the luminescence intensity (relative light unit: RLU) was measured using Lumat^3^ LB 9508 (Berthold Technologies, Bad Wildbad, Germany). Measurements were performed 5 s after reaction with the reagent, and the RLU for 30 s was estimated. The luminescence of each ATP extract was measured twice, and the average of the obtained measurement value was calculated.

### 2.9. Colony Count Assay

After aPDT was completed, the suspension of viable bacteria collected using the above method was diluted by 1/10. Then, 0.1 mL of the diluted suspension was seeded on a BHI agar medium and incubated at 37 °C under a 10% CO_2_ environment for 48 h. After incubation, the number of colonies on the agar medium was counted, and the colony-forming units (CFU) per mL were calculated.

### 2.10. Scanning Electron Microscopy (SEM) Observation of Infected Dentin Plate after aPDT

A specimen of each experimental group after aPDT for SEM observation of the infected dentin plate surface was prepared. The specimen preparation and method for the SEM observation were described above.

### 2.11. Statistical Analysis

The Levene test was conducted to assess the equality of variance for the obtained data. Based on the results of the Levene test, both the ATP and the colony-count assays did not show equal data variances. Hence, the Kruskal–Wallis and Steel-Dwass post hoc tests were used to determine the significant differences among the experimental groups at significant level of 0.05 using BellCurve version 4.04 for Excel (Social Survey Research Information Co., Tokyo, Japan).

## 3. Results

### 3.1. Results of ATP Assay

**Figure 3:**
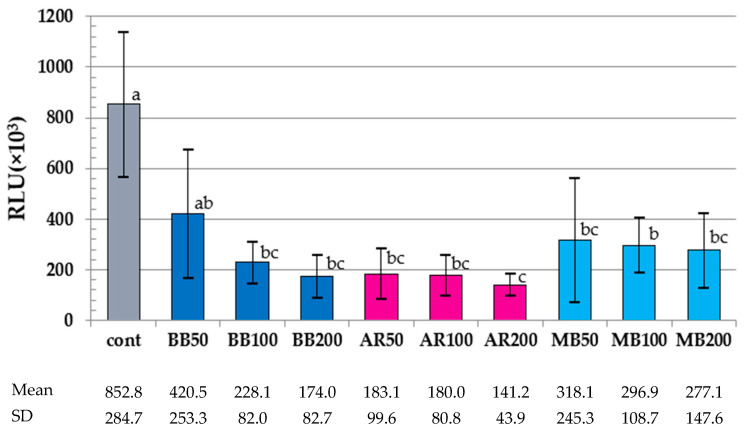
Results of the ATP assay. RLU, relative light units. Different letters indicate a statistically significant difference (*p* < 0.05). Mean and SD are presented as 1/1000 of the actual value.

The results of the ATP assay are summarized in Figure 3. The RLU values of the aPDT groups except for BB50 were significantly lower than that of the control (*p* < 0.05). In the comparative analysis of the RLU values of the aPDT groups, the value of AR200 was significantly lower than that of BB50 or MB100 (*p* < 0.04); however, no differences were observed among the other aPDT groups (*p* > 0.11).

### 3.2. Results of Colony-Count Assay

**Figure 4:**
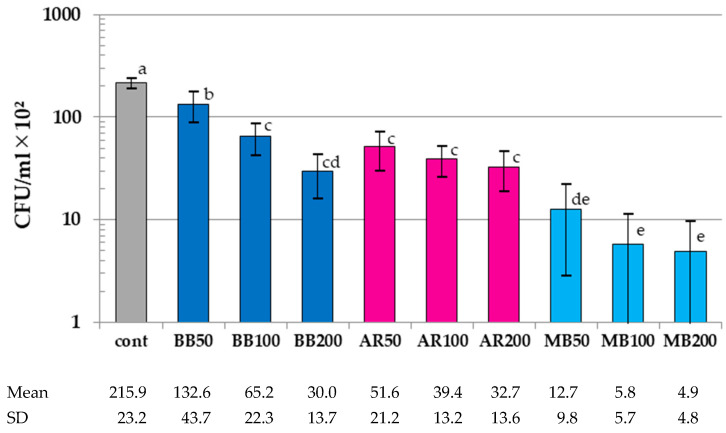
Results of the colony-count assay. CFU, colony-forming units. Different letters indicate a statistically significant difference (*p* < 0.05). Mean and SD are presented as 1/100 of the actual value.

The results of the colony-count assay are summarized in Figure 4. The CFU values of all the aPDT groups were significantly lower than those in the control (*p* < 0.01). When comparing the CFU values of the aPDT groups by photosensitizer, the MB group had significantly lower values than the BB and AR groups, except for BB200 (*p* < 0.01). When comparing the CFU values of the aPDT group by laser irradiation conditions, the value of BB50 was significantly higher than that of BB100 and BB200 in the BB group (*p* < 0.03). On the other hand, there were no significant differences among the experimental groups in both the MB and AR groups (*p* > 0.37).

### 3.3. Scanning Electron Microscope Observations

**Figure 5:**
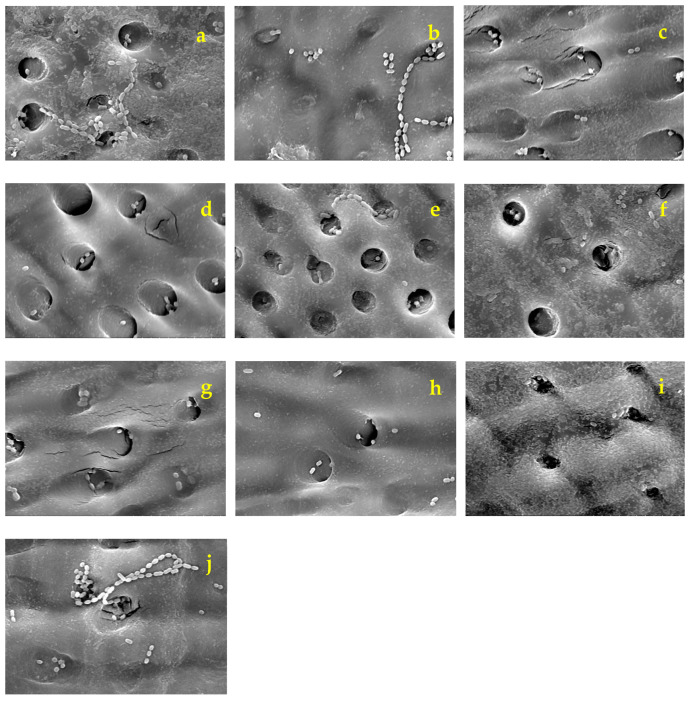
SEM photographs of representative specimens in each experimental group taken after each treatment (×5000). (**a**): BB50, (**b**): BB100, (**c**): BB200, (**d**): AR50, (**e**): AR100, (**f**): AR200, (**g**): MB50, (**h**): MB100, (**i**): MB200, (**j**): cont.

Figure 5 presents SEM images (×5000) of the dentin plates after each aPDT was performed and the PS solution was washed out. Compared with the infected dentin plates assessed before aPDT (Figure 2), a higher number of *S. sobrinus* with broken chains was observed on those evaluated after aPDT application (Figure 5). *S. sobrinus* with chains were observed in relatively high numbers in BB50, BB100, and the control (Figure 5a,b,j). They were even less prevalent in BB200, AR50, AR100, and AR200 (Figure 5c–f). In addition, they were rarely observed in the MB group (Figure 5g–i).

## 4. Discussion

The results of this study indicated that aPDT using a semiconductor laser with a 650 nm wavelength in combination with BB, AR, or MB exhibited high bactericidal efficacy against *S. sobrinus* infection. Furthermore, when the irradiation power and time were regulated to keep the irradiation energy constant, the antimicrobial effect obtained with a higher power and shorter irradiation time tended to be higher than that observed with a lower power and longer irradiation time, although it was not significant under the set conditions in this experiment, and this tendency was more pronounced for BB. This resulted in the rejection of the null hypothesis.

The tendency of the bactericidal effect demonstrated by the colony count was different from that shown by ATP assay depending on the type of PS used in each experimental group. For the bactericidal effects of aPDT with BB and AR, the results of the colony count were almost consistent with those of the ATP assay, whereas the results of the former with MB were quite different from those of the latter. Nagai et al. investigated the bactericidal effect of aPDT involving MB and laser (650 nm) irradiation with the power of 100 mW for 60 s for *S. mutans* and demonstrated a 90% reduction in the CFU value against a 70%–75% decrease in RLU value compared with the control [20]. In the present study, the bactericidal effect of a similar aPDT for *S. sobrinus* exhibited approximately a 97% reduction in the CFU value and approximately 65% in that of the RLU, which is almost consistent with the report by Nagai et al. In an ATP assay, the amount of ATP extracted from each bacterium, even *Streptococci*, is measured as an RLU value. Meanwhile, in a colony-count assay, chained bacteria are counted as a single colony, and the CFU value is calculated. Therefore, the number of viable cells by colony count may be lower than that detected by an ATP assay. Furthermore, even after rinsing PS with PBS from the dentin plate surface after aPDT treatment, the remains of BB and AR were slightly observed on the dentin plate surface. The BB and AR pigments remaining on the dentin plate surface diffused into the bacterial extract solution, resulting in a slight coloration of the solution. It could be assumed that this coloration masked the luminescence of the viable bacteria, and thus, the RLU values were lower than the actual ones.

The results of the colony-count assay indicated that aPDT with MB had a more significant bactericidal effect compared with BB or AR. These outcomes may be attributed to the differences in the penetration of each PS into the bacteria. The cell walls of gram-positive bacteria have a porous structure; therefore, the molecules with the weights of 30,000 to 60,000 Da are permeable into the bacteria [38]. The PS used in the present study can penetrate bacterial cell walls. However, it has been reported that only molecules with a weight of 700 Da or less can enter the cell membrane and cytoplasm [39]. The molecular weights of BB, AR, and MB, which were used in this study, were 825.97, 691.86, and 373.90, respectively. As the molecular weight of MB is almost half of that of BB or AR, MB may penetrate bacterial cell walls more deeply than BB or AR. Hence, it was speculated that MB penetrated not only the cell wall but also the cell membrane and cytoplasm of *S. sobrinus* and that the ROS generated by laser irradiation might have destroyed the bacterial cell resulting in irreversible damage in the bacteria. It has been reported that the number of viable gram-positive bacteria (*Staphylococcus aureus*) decreases with elevated MB concentration when the bacteria in various concentrations were immersed in an MB solution for 60 min in the dark [39]. This report indicated that MB may have possessed bactericidal efficacy by itself; however, no study has suggested these properties of BB and AR. This difference in the bactericidal efficacy of PS itself is probably the reason for the significant difference in the CFU values measured after 48 h between the experimental groups.

Furthermore, assuming the bactericidal effect of aPDT in clinical practice, the penetration of PS into caries lesions is also an important factor. PS is activated when light with a compatible wavelength is absorbed and, firstly, gets an excited singlet form and then transitions to a triplet. The PS at this stage produces various ROS, including free oxygen, under the mechanism called types 1 and 2 [40,41,42]. Yoshii et al. measured the amount of ROS generated by aPDT and reported that aPDT combining BB and laser irradiation at 650 nm wavelength, aPDT combining AR and laser irradiation at the same wavelength, aPDT combining BB and laser irradiation at 940 nm wavelength, and aPDT combining AR and laser irradiation at the same wavelength were 152.4 ± 32.5, 121.1 ± 18.0, 130.0 ± 22.5, and 139.6 ± 15.7, respectively [21]. Although singlet oxygen exerts a high bactericidal effect due to extremely strong oxidative capacity, its diffusion depth is very shallow (10–55 nm) due to an extremely short expression time (10–320 ns) [17,43]. Hence, aPDT may be effective at a peripheral area near PS, whereas its effect may be diminished at a more distant area. Considering these characteristics of aPDT, it is desirable for PS to penetrate sufficiently into caries lesions to sterilize cariogenic bacteria in infected dentin by aPDT.

First, the total energy dose was set to 6 J, referring to the laser irradiation conditions used in a series of studies in our laboratory by Nagai et al. [20] and Yoshii et al. [21]. Next, three irradiation conditions were set so that the total energy dose was 6 J irradiation at an output of 50 mW for 120 s, 100 mW for 60 s, and 200 mW for 30 s. The significance of these three conditions was to investigate the effect of setting the output and time at a low power for a long time, high power for a short time, and intermediate output and time on the antibacterial effect of aPDT. The results of our study indicated that short-time laser irradiation at a high power tended to exert a more significant bactericidal effect than long-time laser irradiation at a low power, and this tendency was more pronounced in BB. Mark et al. measured the amount of ROS generated over time during laser irradiation in PDT, where the irradiation power and time varied, so that the amount of irradiation energy remained constant. They reported that the final total amount of ROS was higher with long-time irradiation at a low power, whereas the ROS generation rate tended to be higher with short-time irradiation at a high power [44]. Yamamoto et al. also evaluated the amount of ROS during aPDT and reported a similar tendency [45]. Although these are reports on PDT-targeting cells, the use of ROS is identical to aPDT-targeting bacteria, which was performed in our study. Therefore, as the maximum concentration of ROS is more important for the assessment of bactericidal effects than its total amount and a threshold may exist for destroying bacteria with cell walls, the experimental results indicated that the high-power short-time irradiation tended to have stronger bactericidal effect when the energy level was constant. The results of the present study can potentially reduce the burden on patients in clinical applications, as high-power short-time irradiation leads to a shorter time for aPDT.

SEM observation of the dentin plate surface showed that more *S. sobrinus* with broken chains were detected after aPDT and that the number of *S. sobrinus* specimens with broken chains tended to increase with increasing laser power in BB and AR. This phenomenon of broken chains may be induced by cell wall injury as *Streptococci* are connected to each other by their cell walls. Hence, higher concentrations of ROS generated by laser irradiation with high power at a short time are expected to be more effective in breaking bacterial cell walls. The applied aPDT with MB showed fewer residual chains regardless of laser power. These results may be attributed to the efficient penetration of MB into bacterial cell walls and the sufficient effect of aPDT involving low-power laser irradiation.

In the present study, aPDT with MB exhibited a significant bactericidal effect, which is consistent with previous reports. However, some studies showed that aPDT with MB exerted adverse effects associated with gingival fibroblasts and osteoblasts [46,47]. Meanwhile, the safety of BB and AR may be higher than that of MB, as they are used as dyes for caries detection. Further studies are warranted to determine the conditions for laser irradiation of aPDT with a more significant bactericidal effect and to confirm the toxicity of aPDT to neighboring tissues such as gingiva and pulp. As a next project, we will search for semiconductor laser irradiation conditions that enhance the bactericidal effect of cariogenic bacteria in aPDT using biosafety BB and AR.

In this study, dentin infection plates were prepared using bovine teeth, but the bacterial invasion by centrifugation into the dentin tubules expanded by phosphoric acid etching was attempted, which is different in aspect from actual human carious dentin. In particular, to mimic softened dentin, the formation of softened dentin by collagenase released by the cariogenic bacteria is necessary with a longer incubation period of the bacteria. This point should be improved in future studies. In addition, the method of preparing bacterial suspensions by detaching viable bacteria from dentin plates requires further refinement. In this study, ultrasonic vibration was used to detach the bacteria from the dentin plates, but the bacteria tended to remain in the plates if the vibration was too short and tended to die if the vibration was too long. In preliminary experiments, the time for applying ultrasonic vibration was examined to determine just the right amount of time, but SEM observation alone is not considered sufficient. In the future, it will be necessary to further investigate a safe and reliable method of detaching viable bacteria from dentin plates.

## 5. Conclusions

Using aPDT combining a 650 nm wavelength semiconductor laser with BB, AR, or MB, showed a remarkable bactericidal effect on *S. sobrinus*-infected dentin plates, especially when used in combination with MB. When irradiation time and power were varied to keep the irradiation energy constant, no significant difference in irradiation conditions was observed in the AR and MB groups, whereas the BB group showed a higher bactericidal effect when irradiated at a higher power for a shorter period. In conclusion, although this study was limited to in vitro studies using infected dentin plates, it was suggested that the bactericidal effect of aPDT on *S. sobrinus* was influenced by the type of photosensitizer and laser irradiation conditions.

## Figures and Tables

**Table 1:** Code for each aPDT experimental group.

Laser Irradiation Protocol	Photo Sensitizer		
	BB	AR	MB
50 mW × 120 s	BB50	AR50	MB50
100 mW × 60 s	BB100	AR100	MB100
200 mW × 30 s	BB200	AR200	MB200

## Data Availability

The data presented in this study are available on request from corresponding author.
